# Supplementary material for: Polarization of HIV-1- and CMV-Specific IL-17-Producing T Cells among People with HIV under Antiretroviral Therapy with Cannabis and/or Cocaine Usage
Source: Pharmaceuticals (Basel). 2024 Apr 6;17(4):465. doi: 10.3390/ph17040465 (PMC11054529; doi:10.3390/ph17040465)
Supplement: Supplementary file 1 [file pharmaceuticals-17-00465-s001.zip › pharmaceuticals-2716799-supplementary.pdf]

Table S1. Statiscal Analyses

**Parameter**	
**Table Analyzed**	
	HIV+ vs HIV+ DU
Mann Whitney test	
P value	0,7374
Exact or approximate P value?	Gaussian Approximation
P value summary	ns
Are medians signif. different? (P < 0.05	No
One- or two-tailed P value?	Two-tailed
Sum of ranks in column E,F	194 , 472
Mann-Whitney U	121
**Table Analyzed**	
	HIV+ vs HIV+ DU
Mann Whitney test	
P value	0,1198
Exact or approximate P value?	Gaussian Approximation
P value summary	ns
Are medians signif. different? (P < 0.05	No
One- or two-tailed P value?	Two-tailed
Sum of ranks in column E,F	125 , 505
Mann-Whitney U	80,0
**Table Analyzed**	
	HIV+ vs HIV+DU
Mann Whitney test	
P value	0,3287
Exact or approximate P value?	Gaussian Approximation
P value summary	ns
Are medians signif. different? (P < 0.05	No
One- or two-tailed P value?	Two-tailed
Sum of ranks in column E,F	174 , 568
Mann-Whitney U	119
**Parameter**	
**Table Analyzed**	

	HIV+<br>vs<br>HIV+ DU
Mann Whitney test	
P value	0,9256
Exact or approximate P value?	Gaussian Approximation
P value summary	ns
Are medians signif. different? (P < 0.05	No
One- or two-tailed P value?	Two-tailed
Sum of ranks in column E,F	188 , 478
Mann-Whitney U	127

**Table Analyzed**	
	HIV+<br>vs<br>HIV+ DU
Mann Whitney test	
P value	0,6178
Exact or approximate P value?	Gaussian Approximation
P value summary	ns
Are medians signif. different? (P < 0.05	No
One- or two-tailed P value?	Two-tailed
Sum of ranks in column E,F	154 , 512
Mann-Whitney U	109

**Table Analyzed**	
Column E<br>vs<br>Column F	HIV+<br>vs<br>HIV+DU
Mann Whitney test	
P value	0,2985
Exact or approximate P value?	Gaussian Approximation
P value summary	ns
Are medians signif. different? (P < 0.05	No
One- or two-tailed P value?	Two-tailed
Sum of ranks in column E,F	217 , 486
Mann-Whitney U	108

Parameter

**Table Analyzed**	
	HIV+<br>vs<br>HIV+ DU

Mann Whitney test	
P value	0,7622
Exact or approximate P value?	Gaussian Approximation
P value summary	ns
Are medians signif. different? (P < 0.05	No
One- or two-tailed P value?	Two-tailed
Sum of ranks in column E,F	176 , 490
Mann-Whitney U	121
**Table Analyzed**	
	HIV+
	vs
	HIV+DU
Mann Whitney test	
P value	0,0376
Exact or approximate P value?	Gaussian Approximation
P value summary	*
Are medians signif. different? (P < 0.05	Yes
One- or two-tailed P value?	Two-tailed
Sum of ranks in column E,F	116 , 550
Mann-Whitney U	71,0
**Table Analyzed**	
Column E	HIV+
vs	vs
Column F	HIV+DU
Mann Whitney test	
P value	0,2985
Exact or approximate P value?	Gaussian Approximation
P value summary	ns
Are medians signif. different? (P < 0.05	No
One- or two-tailed P value?	Two-tailed
Sum of ranks in column E,F	217 , 486
Mann-Whitney U	108

**Parameter**	
**Table Analyzed**	
	HIV+
	vs
	HIV+ DU

Mann Whitney test	
P value	0.0542
Exact or approximate P value?	Gaussian Approximation
P value summary	ns
Are medians signif. different? (P < 0.05	No
One- or two-tailed P value?	Two-tailed
Sum of ranks in column A,B	239 , 465
Mann-Whitney U	86.5

**Table Analyzed**	
	HIV+
	vs
	HIV+ DU
Mann Whitney test	
P value	0.0275
Exact or approximate P value?	Gaussian Approximation
P value summary	*
Are medians signif. different? (P < 0.05	Yes
One- or two-tailed P value?	Two-tailed
Sum of ranks in column A,B	118 , 443
Mann-Whitney U	63

**Table Analyzed**	
	HIV+
	vs
	HIV+ DU
Mann Whitney test	
P value	0,4716
Exact or approximate P value?	Gaussian Approximation
P value summary	ns
Are medians signif. different? (P < 0.05	No
One- or two-tailed P value?	Two-tailed
Sum of ranks in column A,B	169 , 497
Mann-Whitney U	114

**Parameter**	
**Table Analyzed**	
	HIV+
	vs
	HIV+ DU
Mann Whitney test	

P value	0,5662
Exact or approximate P value?	Gaussian Approximation
P value summary	ns
Are medians signif. different? (P < 0.05	No
One- or two-tailed P value?	Two-tailed
Sum of ranks in column A,B	151 , 515
Mann-Whitney U	106

**Table Analyzed**	
	HIV+
	vs
	HIV+DU
Mann Whitney test	
P value	0,2220
Exact or approximate P value?	Gaussian Approximation
P value summary	ns
Are medians signif. different? (P < 0.05	No
One- or two-tailed P value?	Two-tailed
Sum of ranks in column A,B	155 , 548
Mann-Whitney U	100

**Table Analyzed**	
	HIV+
	vs
	HIV+ DU
Mann Whitney test	
P value	0,2669
Exact or approximate P value?	Gaussian Approximation
P value summary	ns
Are medians signif. different? (P < 0.05	No
One- or two-tailed P value?	Two-tailed
Sum of ranks in column A,B	141 , 526
Mann-Whitney U	95,5

**Parameter**	
**Table Analyzed**	
	HIV+
	vs
	HIV+ DU
Mann Whitney test	
P value	0,2054
Exact or approximate P value?	Gaussian Approximation

P value summary	ns
Are medians signif. different? (P < 0.05	No
One- or two-tailed P value?	Two-tailed
Sum of ranks in column A,B	153 , 551
Mann-Whitney U	97,5
**Table Analyzed**	
	HIV+
	vs
	HIV+ DU
Mann Whitney test	
P value	0,9800
Exact or approximate P value?	Gaussian Approximation
P value summary	ns
Are medians signif. different? (P < 0.05	No
One- or two-tailed P value?	Two-tailed
Sum of ranks in column A,B	191 , 512
Mann-Whitney U	134
**Table Analyzed**	
	HIV+
	vs
	HIV+ DU
Mann Whitney test	
P value	0,3223
Exact or approximate P value?	Gaussian Approximation
P value summary	ns
Are medians signif. different? (P < 0.05	No
One- or two-tailed P value?	Two-tailed
Sum of ranks in column A,B	162 , 541
Mann-Whitney U	107

HIV Stimulation

CD4 IFN-y		
HIV+	HIV+	HIV+
vs	vs	vs
HIV+Can	HIV+Coc	HIV+Coc
0.4955	0.5849	0.5849
Gaussian Approximation	Gaussian Approximation	Gaussian Approximation
ns	ns	ns
No	No	No
Two-tailed	Two-tailed	Two-tailed
108 , 82	95.5 , 57.5	95.5 , 57.5
37	29.5	29.5

CD4 IL-17		
HIV+	HIV+	HIV+
vs	vs	vs
HIV+Can	HIV+Coc	HIV+Can+Coc
0.5274	0.3677	0.0296
Gaussian Approximation	Gaussian Approximation	Gaussian Approximation
ns	ns	*
No	No	Yes
Two-tailed	Two-tailed	Two-tailed
83.5 , 107	69 , 67	62.5 , 109
38.5	24	17.5

CD4 IFN-Y/IL-17		
HIV+	HIV+	HIV+
vs	vs	vs
HIV+Can	HIV+Coc	HIV+Can+Coc
0,9544	0,5552	0,5184
Gaussian Approximation	Gaussian Approximation	Gaussian Approximation
ns	ns	ns
No	No	No
Two-tailed	Two-tailed	Two-tailed
99 , 91	85 , 68	98 , 112
44,0	30,0	43,0

CMV Stimulation

CD4 IFN-y

HIV+ vs HIV+Can	HIV+ vs HIV+Coc	HIV+ vs HIV+Can+Coc
0,9662	0,7184	0,9684
Gaussian Approximation	Gaussian Approximation	Gaussian Approximation
ns	ns	ns
No	No	No
Two-tailed	Two-tailed	Two-tailed
100 , 90	94 , 59	104 , 106
45,0	31,0	49,0

CD4 IL-17

HIV+ vs HIV+Can	HIV+ vs HIV+Coc	HIV+ vs HIV+Can+Coc
0,6039	0,2938	0,8046
Gaussian Approximation	Gaussian Approximation	Gaussian Approximation
ns	ns	ns
No	No	No
Two-tailed	Two-tailed	Two-tailed
84 , 106	67 , 69	93 , 97
39,0	22,0	42,0

CD4 IFN-Y/IL-17

HIV+ vs HIV+Can	HIV+ vs HIV+Coc	HIV+ vs HIV+Can+Coc
0,3935	0,9179	0,1206
Gaussian Approximation	Gaussian Approximation	Gaussian Approximation
ns	ns	ns
No	No	No
Two-tailed	Two-tailed	Two-tailed
116 , 94.5	88.5 , 64.5	123 , 87
39,5	33,5	32,0

SEB Stimulation

CD4 IFN-y

HIV+ vs HIV+Can	HIV+ vs HIV+Coc	HIV+ vs HIV+Can+Coc

0,5950	0,4321	0,4480
Gaussian Approximation	Gaussian Approximation	Gaussian Approximation
ns	ns	ns
No	No	No
Two-tailed	Two-tailed	Two-tailed
93 , 97	98.5 , 54.5	94.5 , 116
38,0	26,5	39,5

CD4 IL-17

HIV+ vs HIV+Can	HIV+ vs HIV+Coc	HIV+ vs HIV+Can+Coc
0.0142	0,8540	0,1267
Gaussian Approximation	Gaussian Approximation	Gaussian Approximation
**	ns	ns
Yes	No	No
Two-tailed	Two-tailed	Two-tailed
56 , 134	75 , 61	75 , 115
11,0	30,0	30,0

CD4 IFN-Y/IL-17

HIV+ vs HIV+Can	HIV+ vs HIV+Coc	HIV+ vs HIV+Can+Coc
0,3935	0,9179	0,1206
Gaussian Approximation	Gaussian Approximation	Gaussian Approximation
ns	ns	ns
No	No	No
Two-tailed	Two-tailed	Two-tailed
116 , 94.5	88.5 , 64.5	123 , 87
39,5	33,5	32,0

HIV Stimulation

CD8 IFN-Y

HIV+ vs HIV+Can	HIV+ vs HIV+Coc	HIV+ vs HIV+Can+Coc

0.1593	0.4281	0.0549
Gaussian Approximation	Gaussian Approximation	Gaussian Approximation
ns	ns	ns
No	No	No
Two-tailed	Two-tailed	Two-tailed
123 , 87.5	98 , 55	128 , 82
32.5	27	27

CD8 IL-17

HIV+	HIV+	HIV+
vs	vs	vs
HIV+Can	HIV+Coc	HIV+Can+Coc
0.3278	0.2036	0.0296
Gaussian Approximation	Gaussian Approximation	Gaussian Approximation
ns	ns	*
No	No	Yes
Two-tailed	Two-tailed	Two-tailed
94 , 116	79 , 74	79 , 131
39	24	24

CD8 IFN-y/IL-17

HIV+	HIV+	HIV+
vs	vs	vs
HIV+ can	HIV+ coc	HIV+Can+Coc
0,8712	0,2031	0,8712
Gaussian Approximation	Gaussian Approximation	Gaussian Approximation
ns	ns	ns
No	No	No
Two-tailed	Two-tailed	Two-tailed
103 , 107	79 , 74	103 , 107
48,0	24,0	48,0

CMV Stimulation

CD8 IFN-Y

HIV+	HIV+	HIV+
vs	vs	vs
HIV+Can	HIV+Coc	HIV+Can+Coc

0,7626	0,3280	0,7117
Gaussian Approximation	Gaussian Approximation	Gaussian Approximation
ns	ns	ns
No	No	No
Two-tailed	Two-tailed	Two-tailed
86 , 104	67 , 69	81 , 90
41,0	22,0	36,0

CD8 IL-17

HIV+ vs HIV+Can	HIV+ vs HIV+Coc	HIV+ vs HIV+Can+Coc
0,5005	0.0426	0,9012
Gaussian Approximation	Gaussian Approximation	Gaussian Approximation
ns	*	ns
No	Yes	No
Two-tailed	Two-tailed	Two-tailed
96 , 114	66 , 87	103 , 107
41,0	11,0	48,0

CD8 IFN-y/IL-17

HIV+ vs HIV+Can	HIV+ vs HIV+Coc	HIV+ vs HIV+Can+Coc
0,2988	0,3032	0,5274
Gaussian Approximation	Gaussian Approximation	Gaussian Approximation
ns	ns	ns
No	No	No
Two-tailed	Two-tailed	Two-tailed
79 , 111	68 , 68	83.5 , 107
34,0	23,0	38,5

SEB Stimulation

CD8 IFN-Y

HIV+ vs HIV+Can	HIV+ vs HIV+Coc	HIV+ vs HIV+Can+Coc
0,3256	0,1613	0,5199
Gaussian Approximation	Exact	Gaussian Approximation

ns	ns	ns
No	No	No
Two-tailed	Two-tailed	Two-tailed
91.5 , 119	75 , 78	96 , 114
36,5	20,0	41,0

CD8 IL-17

HIV+	HIV+	HIV+
vs	vs	vs
HIV+Can	HIV+Coc	HIV+Can+Coc
0,5843	0,6463	0,9569
Gaussian Approximation	Gaussian Approximation	Gaussian Approximation
ns	ns	ns
No	No	No
Two-tailed	Two-tailed	Two-tailed
110 , 100	86 , 67	105 , 105
45,0	31,0	50,0

CD8 IFN-y/IL-17

HIV+	HIV+	HIV+
vs	vs	vs
HIV+Can	HIV+Coc	HIV+Can+Coc
0,6863	0,2062	0,4665
Gaussian Approximation	Gaussian Approximation	Gaussian Approximation
ns	ns	ns
No	No	No
Two-tailed	Two-tailed	Two-tailed
99.5 , 111	77 , 76	95.5 , 115
44,5	22,0	40,5

Parameter		HIV S
Table Analyzed		C
	HIV+<br>vs<br>HIV+ DU	HIV+<br>vs<br>HIV+Can
Mann Whitney test		
P value	0.0542	0.1593
Exact or approximate P value?	Gaussian Approximation	Gaussian Approximation
P value summary	ns	ns
Are medians signif. different? (P < 0.05)	No	No
One- or two-tailed P value?	Two-tailed	Two-tailed
Sum of ranks in column A,B	239 , 465	123 , 87.5
Mann-Whitney U	86.5	32.5

Table Analyzed		C
	HIV+<br>vs<br>HIV+ DU	HIV+<br>vs<br>HIV+Can
Mann Whitney test		
P value	0.0275	0.3278
Exact or approximate P value?	Gaussian Approximation	Gaussian Approximation
P value summary	*	ns
Are medians signif. different? (P < 0.05)	Yes	No
One- or two-tailed P value?	Two-tailed	Two-tailed
Sum of ranks in column A,B	118 , 443	94 , 116
Mann-Whitney U	63	39

Table Analyzed		CD8
	HIV+<br>vs<br>HIV+ DU	HIV+<br>vs<br>HIV+ can
Mann Whitney test		
P value	0,4716	0,8712
Exact or approximate P value?	Gaussian Approximation	Gaussian Approximation
P value summary	ns	ns
Are medians signif. different? (P < 0.05)	No	No
One- or two-tailed P value?	Two-tailed	Two-tailed
Sum of ranks in column A,B	169 , 497	103 , 107
Mann-Whitney U	114	48,0

Parameter		CMV S
Table Analyzed		C
	HIV+<br>vs<br>HIV+ DU	HIV+<br>vs<br>HIV+Can

Mann Whitney test		
P value	0,5662	0,7626
Exact or approximate P value?	Gaussian Approximation	Gaussian Approximation
P value summary	ns	ns
Are medians signif. different? (P < 0.05)	No	No
One- or two-tailed P value?	Two-tailed	Two-tailed
Sum of ranks in column A,B	151 , 515	86 , 104
Mann-Whitney U	106 41,0	

**Table Analyzed**		C
	HIV+	HIV+
	vs	vs
	HIV+DU	HIV+Can
Mann Whitney test		
P value	0,2220	0,5005
Exact or approximate P value?	Gaussian Approximation	Gaussian Approximation
P value summary	ns	ns
Are medians signif. different? (P < 0.05)	No	No
One- or two-tailed P value?	Two-tailed	Two-tailed
Sum of ranks in column A,B	155 , 548	96 , 114
Mann-Whitney U	100	41,0

**Table Analyzed**		**CD8**
	HIV+	HIV+
	vs	vs
	HIV+ DU	HIV+Can
Mann Whitney test		
P value	0,2669	0,2988
Exact or approximate P value?	Gaussian Approximation	Gaussian Approximation
P value summary	ns	ns
Are medians signif. different? (P < 0.05)	No	No
One- or two-tailed P value?	Two-tailed	Two-tailed
Sum of ranks in column A,B	141 , 526	79 , 111
Mann-Whitney U	95,5	34,0

**Parameter**		**SEB S**
**Table Analyzed**		C
	HIV+	HIV+
	vs	vs
	HIV+ DU	HIV+Can
Mann Whitney test		
P value	0,2054	0,3256
Exact or approximate P value?	Gaussian Approximation	Gaussian Approximation
P value summary	ns	ns

Are medians signif. different? (P < 0.05)	No	No
One- or two-tailed P value?	Two-tailed	Two-tailed
Sum of ranks in column A,B	153 , 551	91.5 , 119
Mann-Whitney U	97,5	36,5

**Table Analyzed**		C
	HIV+ vs HIV+ DU	HIV+ vs HIV+Can
Mann Whitney test		
P value	0,9800	0,5843
Exact or approximate P value?	Gaussian Approximation	Gaussian Approximation
P value summary	ns	ns
Are medians signif. different? (P < 0.05)	No	No
One- or two-tailed P value?	Two-tailed	Two-tailed
Sum of ranks in column A,B	191 , 512	110 , 100
Mann-Whitney U	134	45,0

**Table Analyzed**		**CD8**
	HIV+ vs HIV+ DU	HIV+ vs HIV+Can
Mann Whitney test		
P value	0,3223	0,6863
Exact or approximate P value?	Gaussian Approximation	Gaussian Approximation
P value summary	ns	ns
Are medians signif. different? (P < 0.05)	No	No
One- or two-tailed P value?	Two-tailed	Two-tailed
Sum of ranks in column A,B	162 , 541	99.5 , 111
Mann-Whitney U	107	44,5

timulation

D8 IFN-Y

HIV+	HIV+
vs	vs
HIV+Coc	HIV+Can+Coc
0.4281	0.0549
Gaussian Approximation	Gaussian Approximation
ns	ns
No	No
Two-tailed	Two-tailed
98 , 55	128 , 82
27	27

D8 IL-17

HIV+	HIV+
vs	vs
HIV+Coc	HIV+Can+Coc
0.2036	0.0296
Gaussian Approximation	Gaussian Approximation
ns	*
No	Yes
Two-tailed	Two-tailed
79 , 74	79 , 131
24	24

IFN-y/IL-17

HIV+	HIV+
vs	vs
HIV+ coc	HIV+Can+Coc
0,2031	0,8712
Gaussian Approximation	Gaussian Approximation
ns	ns
No	No
Two-tailed	Two-tailed
79 , 74	103 , 107
24,0	48,0

Stimulation

D8 IFN-Y

HIV+	HIV+
vs	vs
HIV+Coc	HIV+Can+Coc

0,3280	0,7117
Gaussian Approximation	Gaussian Approximation
ns	ns
No	No
Two-tailed	Two-tailed
67 , 69	81 , 90
22,0	36,0

CD8 IL-17

HIV+ vs HIV+Coc	HIV+ vs HIV+Can+Coc
0.0426	0,9012
Gaussian Approximation	Gaussian Approximation
*	ns
Yes	No
Two-tailed	Two-tailed
66 , 87	103 , 107
11,0	48,0

IFN-γ/IL-17

HIV+ vs HIV+Coc	HIV+ vs HIV+Can+Coc
0,3032	0,5274
Gaussian Approximation	Gaussian Approximation
ns	ns
No	No
Two-tailed	Two-tailed
68 , 68	83.5 , 107
23,0	38,5

timulation

CD8 IFN-Y

HIV+ vs HIV+Coc	HIV+ vs HIV+Can+Coc
0,1613	0,5199
Exact	Gaussian Approximation
ns	ns

No	No
Two-tailed	Two-tailed
75 , 78	96 , 114
20,0	41,0

CD8 IL-17

HIV+	HIV+
vs	vs
HIV+Coc	HIV+Can+Coc
0,6463	0,9569
Gaussian Approximation	Gaussian Approximation
ns	ns
No	No
Two-tailed	Two-tailed
86 , 67	105 , 105
31,0	50,0

IFN-γ/IL-17

HIV+	HIV+
vs	vs
HIV+Coc	HIV+Can+Coc
0,2062	0,4665
Gaussian Approximation	Gaussian Approximation
ns	ns
No	No
Two-tailed	Two-tailed
77 , 76	95.5 , 115
22,0	40,5
